# Association of cerebrospinal fluid anti-Sm antibodies with acute confusional state in systemic lupus erythematosus

**DOI:** 10.1186/s13075-014-0450-z

**Published:** 2014-10-02

**Authors:** Shunsei Hirohata, Yuko Sakuma, Tamiko Yanagida, Taku Yoshio

**Affiliations:** Department of Rheumatology and Infectious Diseases, Kitasato University School of Medicine, 1-15-1 Kitasato, Sagamihara, Kanagawa 252-0374 Japan; Department of Internal Medicine, Teikyo University School of Medicine, 2-11-1 Kaga, Itabashi-ku, Tokyo 173-8605 Japan; Division of Rheumatology and Clinical Immunology, Jichi Medical University, 3311‐1 Yakushiji, Shimotsuke‐shi, Tochigi 329-0498 Japan

## Abstract

**Introduction:**

Neuropsychiatric manifestation in systemic lupus erythematosus (NPSLE) is one of the most serious complications of the disease. Previous studies revealed the strong association between serum anti-Sm and organic brain syndrome, consisting mainly of acute confusional state (ACS) of diffuse psychiatric/neuropsychological syndromes (diffuse NPSLE). However, the precise mechanism by which anti-Sm causes diffuse NPSLE remains unclear. Of note, recent studies demonstrated that anti-U1 RNP antibodies (anti-RNP) in cerebrospinal fluid (CSF) are associated with NPSLE. The present study was designed to explore the association of anti-Sm antibodies in CSF with NPSLE.

**Methods:**

Paired serum and CSF specimens were obtained from 72 patients with NPSLE (49 with diffuse NPSLE, 23 with neurological syndromes or peripheral neuropathy (focal NPSLE) and from 22 control patients with non-SLE neurological diseases. Sera were also obtained from 41 patients with active SLE without neuropsychiatric manifestations (non-NPSLE). Anti-Sm and anti-RNP were measured by enzyme-linked immunosorbent assay (ELISA). Blood-brain barrier (BBB) function and intrathecal anti-Sm production were evaluated by Q albumin and CSF anti-Sm index, respectively. Binding of anti-Sm to neuroblastoma cell lines SK-N-MC and Neuro2a was examined by flow cytometry and by cell ELISA.

**Results:**

Anti-Sm and anti-RNP in CSF and sera were elevated in NPSLE compared with non-SLE control. CSF anti-Sm, but not CSF anti-RNP, was significantly elevated in ACS compared with non-ACS diffuse NPSLE or with focal NPSLE. By contrast, there were no significant differences in serum anti-Sm or anti-RNP among subsets of NPSLE and non-NPSLE. Whereas there were no significant differences in CSF anti-Sm index, Q albumin was elevated in ACS compared with non-ACS or with focal NPSLE. Notably, CSF anti-Sm was correlated with Q albumin (r = 0.2373, *P* = 0.0447) or with serum anti-Sm (r = 0.7185, *P* <0.0001) in 72 patients with NPSLE. Finally, monoclonal anti-Sm and purified human anti-Sm bound to the surface of SK-N-MC and Neuro2a.

**Conclusions:**

These results demonstrate that the elevation of CSF anti-Sm through transudation from systemic circulation due to damaged BBB plays a critical role in the pathogenesis of ACS. More importantly, the data indicate that anti-Sm is yet another autoantibody with presumed neural toxicity, but might not be the last.

## Introduction

Neuropsychiatric manifestation in systemic lupus erythematosus (NPSLE) is one of the most serious complications of the disease [1,2]. The role of anti-neuronal antibodies in the pathogenesis of NPSLE has been appreciated since Bluestein *et al*. demonstrated that immunoglobulin G (IgG) anti-neuronal antibodies were present in much higher concentrations in the cerebrospinal fluid (CSF) from patients with active NPSLE [3]. Of interest, CSF IgG anti-neuronal antibodies were found to be significantly elevated in patients with diffuse psychiatric/neuropsychological syndromes (diffuse NPSLE) compared with neurologic syndromes (focal NPSLE) [4]. However, the epitopes to which CSF anti-neuronal antibodies were directed have not been fully delineated.

N-methyl-D-aspartate (NMDA) receptors are one of the glutamate receptor families and its stimulation has been shown to cause excitatory synaptic transmission in the central nervous system (CNS) [5]. DeGiorgio *et al*. demonstrated that a subset of murine anti-DNA antibodies cross-reacts with a sequence within the NMDA receptor subunit NR2 [6]. Furthermore, injection into mouse brain of such cross-reactive anti-DNA antibodies purified from serum or CSF of an SLE patient with progressive cognitive impairment caused neuronal damage [6]. Notably, the presence of such cross-reactive anti-DNA antibodies in the serum compartment alone could not result in brain damage, which also requires a breakdown of the blood-brain barrier (BBB) to allow such autoantibodies to enter the CNS [7]. Accordingly, we showed that CSF anti-NMDA receptor subunit NR2 (anti-NR2) antibodies, but not serum anti-NR2, were closely associated with diffuse NPSLE [8].

On the other hand, the association of serum anti-Sm antibodies with CNS involvement in SLE has been suggested [9,10]. In addition, a strong association was found between serum anti-Sm and organic brain syndrome, consisting mainly of acute confusional state (ACS) of diffuse NPSLE [11]. However, the precise mechanism by which anti-Sm causes diffuse NPSLE remains unclear. Of note, recent studies have demonstrated that anti-U1 RNP antibodies (anti-RNP) in CSF, but not those in serum, are associated with central neuropsychiatric manifestations in SLE and mixed connected tissue disease (MCTD) [12]. It should be pointed out that anti-Sm and anti-RNP are closely correlated. Thus, the antibody pattern produced by the mice immunized with exogenous U1 small nuclear RNP particles was strikingly similar to that observed in patients with SLE [13]. It is therefore possible that anti-Sm might also be present in CSF from patients with NPSLE. The current studies were therefore undertaken to compare the levels of CSF anti-Sm in patients with various types of NPSLE and non-SLE non-inflammatory neurological disorders.

## Methods

### Patients and samples

Seventy-two patients with SLE were included in the present study. All patients fulfilled the American College of Rheumatology (ACR) 1982 revised criteria for the classification of SLE [14]. Of the 72 NPSLE patients, 49 showed diffuse psychiatric/neuropsychological syndromes (diffuse NPSLE) according to the 1999 ACR definition of NPSLE [15], whereas 23 patients showed CNS manifestations other than diffuse NPSLE, including neurologic syndromes and peripheral nervous system involvements (focal NPSLE) (Table 1). All the patients with NPSLE were hospitalized in Teikyo University Hospital or other correlated hospitals between 2000 and 2008. In addition, serum samples were obtained from 41 patients with active SLE without neuropsychiatric manifestations (non-NPSLE) (age 36.9 ± 14.9 years (mean ± standard deviation (SD))). As non-SLE control, 22 patients with non-SLE non-inflammatory neurologic diseases (7 with cervical spondylitis, 7 with cerebrovascular diseases, 3 with neurodegenerative diseases, 2 with hyperventilation syndrome, 2 with diabetic neuropathy, 1 with headache) were studied. All the 135 patients gave informed consent. This study was approved by the institutional ethics committee of Teikyo University School of Medicine.

**Table 1:** **Profiles of the patients studied**

**Diagnosis**	**No. of patients**	**Gender (male/female)**	**Age (mean ± SD)**
Systemic lupus erythematosus (SLE)	72		
Diffuse NPSLE	49	7/42	38.3 ± 14.4
Acute confusional state	19		
Anxiety disorder	3		
Cognitive dysfunction	8^*^		
Mood disorder	12		
Psychosis	7		
Focal NPSLE	23	4/19	42.0 ± 15.2
Cerebrovascular disease	9		
Demyelinating syndrome	1		
Headache	2		
Movement disorder	2		
Seizure disorder	7		
Aseptic meningitis	1		
Polyneuropathy	1		
Non SLE control	22	21/1	49.4 ± 10.5

^*^Two patients also presented with mood disorder. SD: standard deviation; (NP)SLE: (neuropsychiatric) systemic lupus erythematosus.

CSF specimens were obtained from the patients by lumbar puncture on the same day serum samples were obtained, when neurologists and rheumatologists made the diagnosis of NPSLE in each institution. These samples were kept frozen at −30°C until they were assayed. All assays were performed without knowledge of the diagnosis or clinical presentation. Furthermore, upon entering the present study, the diagnosis of the 72 patients with NPSLE and its classification was reconfirmed by referral to rheumatologists in charge as well as by review of hospital case records in each institution.

### Measurement of ant-Sm, anti-RNP, anti-NMDA receptor subunit NR2 (anti-NR2) and albumin

Anti-Sm and anti-RNP levels in CSF and sera were measured using enzyme-linked immunosorbent assay (ELISA) kits, MESACUP™-3 test Sm and MESACUP™-2 test RNP (MBL, Nagano, Japan). Arbitrary unit was designated according to the manufacturer’s instructions. Anti-NR2 in sera and CSF was determined by specific ELISA using the highly purified synthetic 10 amino-acid peptide DWEYSVWLSN [6], conjugated to human serum albumin (HSA) as previously described [8]. Albumin levels in CSF and sera were measured by ELISA using the Human Albumin ELISA Quantitation Set (Bethyl Laboratories, Montgomery, TX, USA).

### Evaluation of blood-brain barrier function and intrathecal synthesis of anti-Sm or anti-RNP

Blood-brain barrier (BBB) function and intrathecal synthesis of anti-Sm or anti-RNP were evaluated by Q albumin (CSF albumin × 1,000/serum albumin) and by CSF anti-Sm or anti-RNP index ([CSF anti-Sm or anti-RNP × serum albumin]/[serum anti-Sm or anti-RNP × CSF albumin]), respectively, as previously described [16].

### Immunofluorescence staining and analysis

To explore whether anti-Sm binds neuronal cells, the binding of murine monoclonal anti-Sm (murine IgG3, Abcam, Tokyo, Japan) to human neuroblastoma cell line SK-N-MC cells and murine neuroblastoma cell line Neuro2a cells was examined. Briefly, after being fixed with 1% paraformaldehyde in phosphate-buffered saline (PBS) (pH 7.2) for 5 minutes at 37°C, the cells were washed with 2% normal human serum in PBS and 0.1% sodium azide (staining buffer). The cells were then reacted with monoclonal anti-Sm or control murine IgG3 (Abcam) (5 μg /ml) at 4°C for 30 minutes. After 3 washes with staining buffer, the cells were counterstained with fluorescein isothiocyanate-conjugated F(ab’)_2_ fragments of goat anti-mouse IgG (Cappel Laboratories, Cochranville, PA, USA). After staining, the cells were treated in saline with 50 μg/ml propidium iodide (PI; Sigma-Aldrich, St. Louis, MO, USA) for more than 5 minutes at room temperature, followed by analysis using Cell Lab Quanta SC (Beckman Coulter, Miami, FL, USA). The gating threshold for PI staining to identify viable cells was determined using cells without PI staining [17]. The percentages of cells that were stained positively for anti-Sm were determined in relation to the percentage of staining with control murine IgG3. The density of staining was expressed as the change in mean fluorescence intensity (MFI) for staining with anti-Sm, which was calculated by subtracting the MFI of staining with control IgG3.

### Cell ELISA

To further confirm the binding of anti-Sm to neuronal cells, a cell ELISA was carried out using human neuroblastoma cell line SK-N-MC as previously described [4]. Briefly, SK-N-MC cells were seeded at a density of 5 × 10^4^ per well in wells of a flat-bottomed 96-well tissue culture plate for 48 hours, after which the cells were fixed with 1% paraformaldehyde in PBS for 5 minutes at 37°C. After 3 washes with PBS containing 0.05% Tween 20, the wells were reacted with 50 μl of murine monoclonal anti-Sm (5 μg/ml) or human anti-Sm (50 μg/ml) purified from IgG fraction of serum of an SLE patient using an *N*-hydroxysuccinimide-activated Sepharose HP column (Amersham Biosciences, Uppsala, Sweden) coupled with purified human Sm antigens (ImmunoVision, Springdale, AR, USA) according to the manufacturer’s instructions. Murine monoclonal IgG3 (5 μg/ml) or control human IgG (50 μg/ml) purified from serum of a normal healthy individual was used as control. After incubation for 1 hour at 37°C, bound IgG was detected with peroxidase-conjugated F(ab’)_2_ fragments of goat anti-mouse IgG or anti-human IgG (Cappel Laboratories, West Chester, PA, USA), as previously described [4]. The results are expressed by the absorbance at 492 nm (OD492).

### Statistical analysis

Comparisons among 3 groups and those between 2 groups were carried out by Kruskal-Wallis test with Dunn’s multiple comparison test and by Mann-Whitney’s *U* test, respectively, using GraphPad Prism 4 (Windows ver. 4.03; GraphPad Software, Inc., San Diego, CA, USA).

## Results

### CSF anti-Sm and anti-RNP in NPSLE

**Figure 1:**
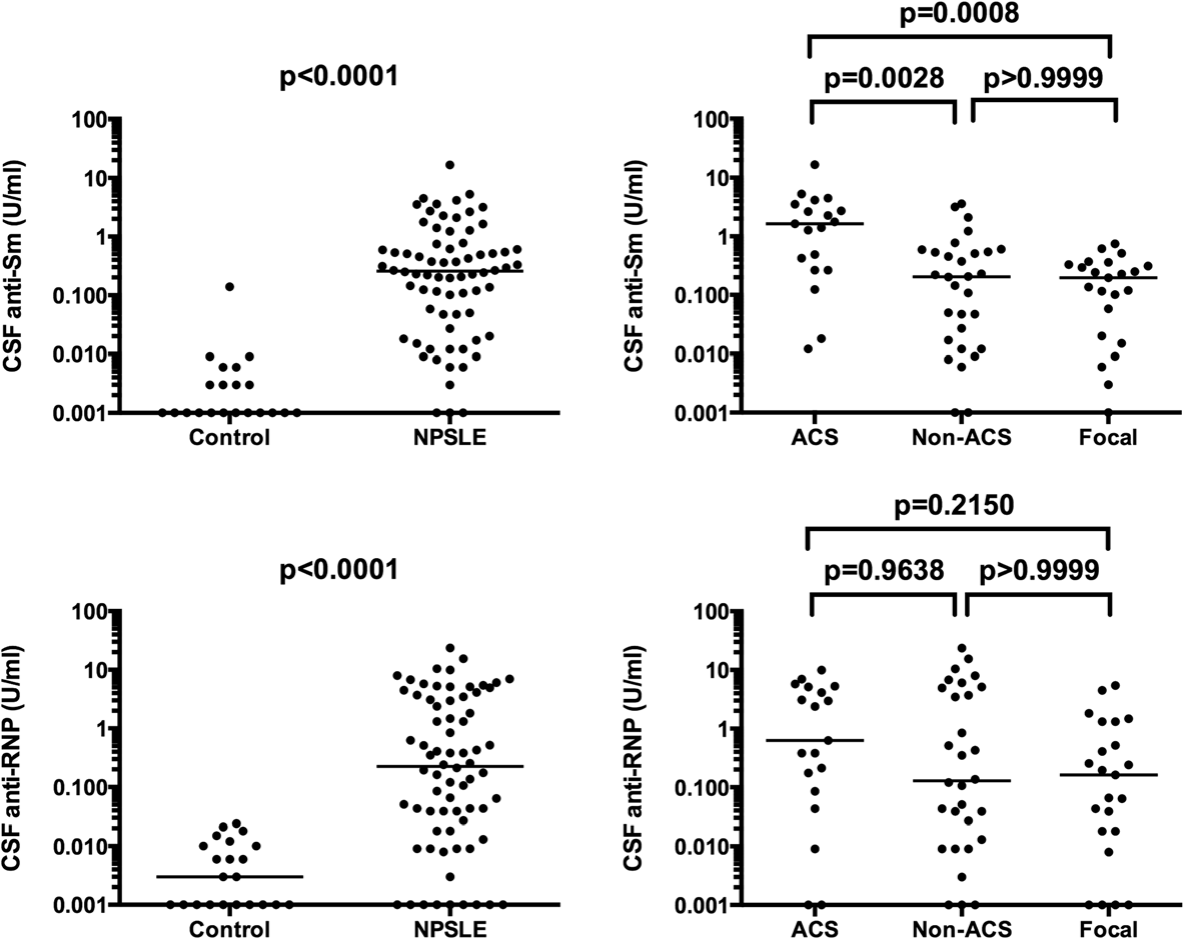
**Cerebrospinal fluid (CSF) anti-Sm and anti-RNP in NPSLE.** CSF anti-Sm levels were 0.009 ± 0.029 (mean ± SD) in non-SLE neurological control and 0.977 ± 2.221 in NPSLE: 2.604 ± 3.774 in acute confusional state (ACS), 0.528 ± 0.897 in non-ACS diffuse NPSLE, 0.219 ± 0.205 in focal NPSLE. CSF anti-RNP levels were 0.007 ± 0.007 (mean ± SD) in non-SLE neurological control and 2.164 ± 3.984 in NPSLE: 2.506 ± 2.967 in ACS, 3.010 ± 5.431 in non-ACS diffuse NPSLE, 0.778 ± 1.437 in focal NPSLE. Horizontal lines indicate median. NPSLE: neuropsychiatric systemic lupus erythematosus; SD: standard deviation.

Anti-Sm and anti-RNP in CSF were determined by ELISA. Anti-Sm and anti-RNP in CSF from 22 control patients were 0.009 ± 0.029 U/ml and 0.007 ± 0.007 U/ml (mean ± SD), respectively. Both anti-Sm and anti-RNP in CSF were significantly elevated in NPSLE compared with non-SLE control. Among subsets of NPSLE, CSF anti-Sm was significantly elevated in ACS compared with non-ACS diffuse NPSLE (*P* = 0.0028) or with focal NPSLE (*P* = 0.0008) (Figure 1). By contrast, there were no significant differences in CSF anti-RNP among the 3 groups of NPSLE. These results indicate that the elevation of CSF anti-Sm, but not that of anti-RNP, is associated with the development of ACS.

### Serum anti-Sm and anti-RNP in NPSLE

**Figure 2:**
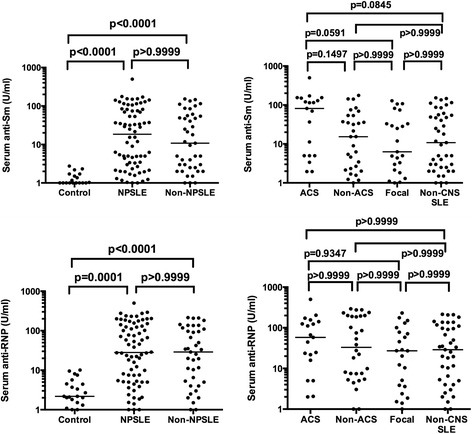
**Serum anti-Sm and anti-RNP in NPSLE.** Serum anti-Sm levels were 1.221 ± 0.644 (mean ± SD) in non-SLE neurological control, 49.94 ± 75.04 in NPSLE and 35.76 ± 45.41 in non-NPSLE: 96.39 ± 116.4 in acute confusional state (ACS), 35.61 ± 47.1 in non-ACS diffuse NPSLE, 30.24 ± 40.22 in focal NPSLE. Serum anti-RNP levels were 3.704 ± 2.855 (mean ± SD) in non-SLE neurological control, 80.05 ± 97.82 in NPSLE and 59.55 ± 67.37 in non-NPSLE: 94.79 ± 117.7 in ACS, 92.41 ± 103.4 in non-ACS diffuse NPSLE, 51.76 ± 64.91 in focal NPSLE. Horizontal lines indicate median. NPSLE: neuropsychiatric systemic lupus erythematosus; SD: standard deviation.

We next examined anti-Sm and anti-RNP in sera. Anti-Sm and anti-RNP in sera from 22 control patients were 1.221 ± 0.644 U/ml and 3.704 ± 2.855 U/ml (mean ± SD), respectively. Both anti-Sm and anti-RNP in sera were significantly elevated in NPSLE as well as in non-NPSLE compared with non-SLE control, whereas there were no significant differences between NPSLE and non-NPSLE. Accordingly, there were no significant differences in anti-Sm and anti-RNP in the sera among subsets of NPSLE and non-NPSLE. Although serum anti-Sm appeared to be higher in ACS than in the other 3 groups of SLE, it did not reach statistical significance (Figure 2). These results suggest that the elevation of serum anti-Sm might contribute only little, if any, to the development of ACS.

### Intrathecal production of anti-Sm and anti-RNP in NPSLE

**Figure 3:**
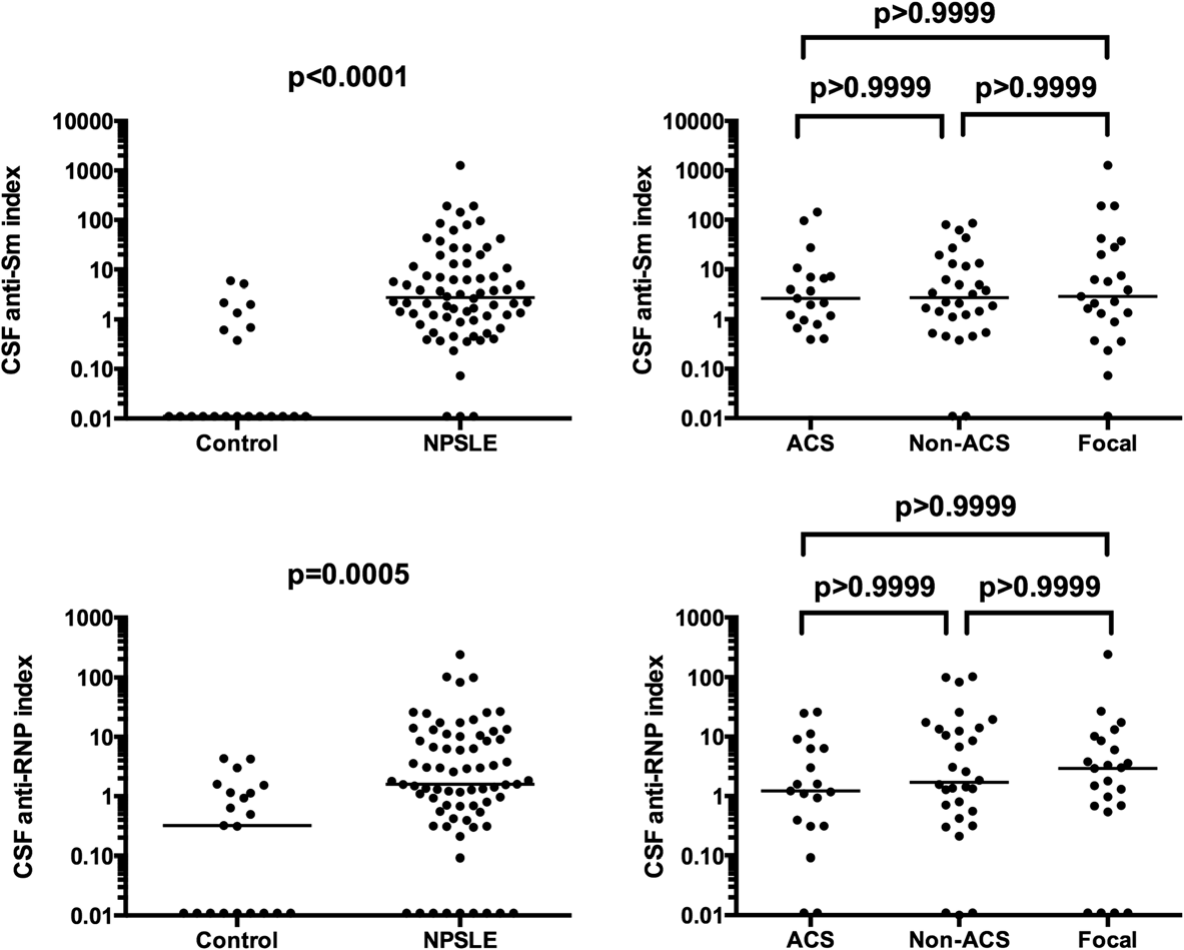
**Intrathecal production of anti-Sm and anti-RNP in NPSLE.** Cerebrospinal fluid (CSF) anti-Sm index was 0.881 ± 1.696 (mean ± SD) in non-SLE neurological control and 35.14 ± 152.3 in NPSLE: 16.72 ± 37.61 in acute confusional state (ACS), 13.21 ± 23.35 in non-ACS diffuse NPSLE, 78.95 ± 264.5 in focal NPSLE. CSF anti-RNP index was 0.941 ± 1.347 (mean ± SD) in non-SLE neurological control and 12.10 ± 33.35 in NPSLE: 5.02 ± 7.89 in ACS, 14.35 ± 28.28 in non-ACS diffuse NPSLE, 15.00 ± 49.25 in focal NPSLE. Horizontal lines indicate median. NPSLE: neuropsychiatric systemic lupus erythematosus; SD: standard deviation.

Previous studies demonstrated that intrathecal immunoglobulin production was increased in NPSLE [18,19]. It is therefore possible that intrathecal production of ant-Sm and anti-RNP might be increased in NPSLE. We next examined CSF anti-Sm or anti-RNP index, which reflects the intrathecal production of these antibodies. CSF anti-Sm and anti-RNP indices in 22 control patients were 0.881 ± 1.696 and 0.941 ± 1.347 (mean ± SD), respectively. Both CSF anti-Sm and anti-RNP indices were significantly elevated in NPSLE compared with non-SLE control. However, there were no significant differences in CSF anti-Sm or anti-RNP index among the 3 groups of NPSLE (Figure 3). These results indicate that the elevation of CSF anti-Sm in ACS cannot be accounted for by the increased production within the CNS.

### Relationship of BBB integrity and serum anti-Sm with CSF anti-Sm in NPSLE

**Figure 4:**
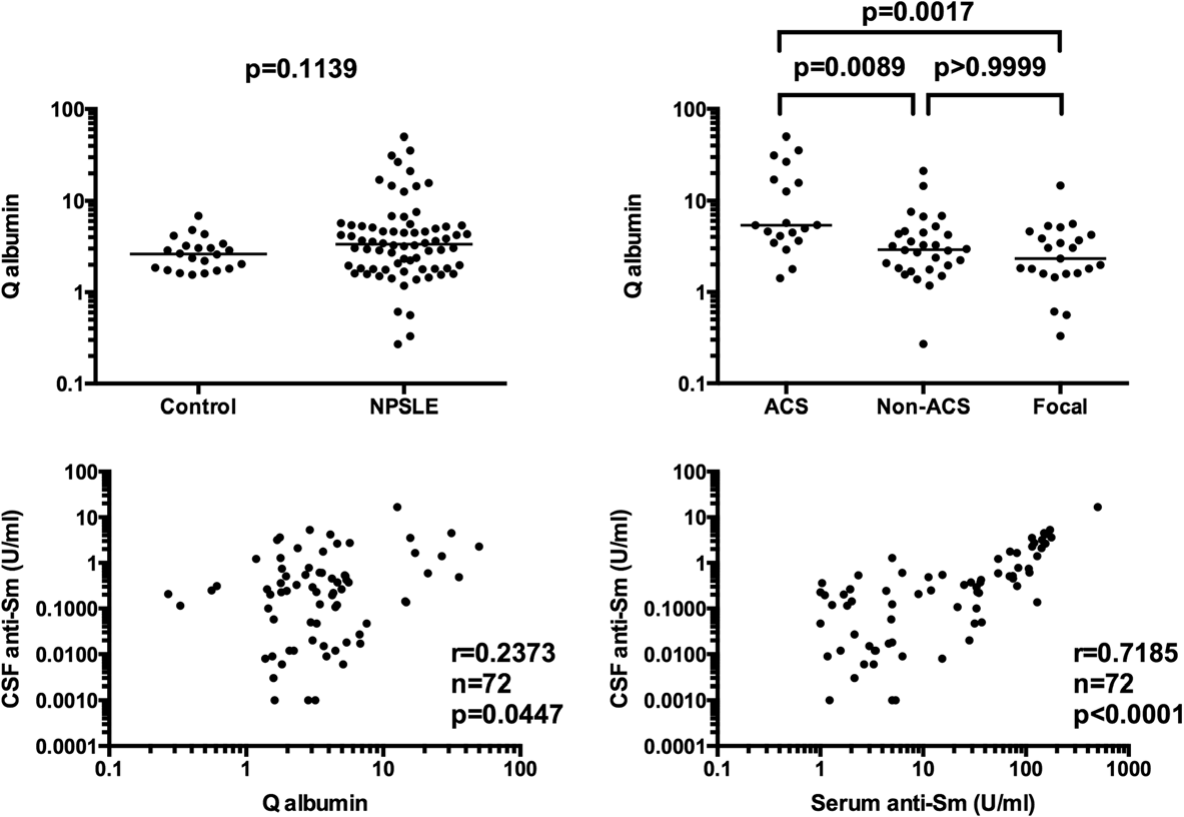
**Relationship of blood-brain barrier integrity and serum anti-Sm with cerebrospinal fluid (CSF) anti-Sm in NPSLE.** In upper panels, Q albumin values were 2.84 ± 1.29 (mean ± SD) in non-SLE neurological control and 6.05 ± 8.56 in NPSLE: 12.48 ± 13.80 in acute confusional state (ACS), 4.14 ± 4.20 in non-ACS diffuse NPSLE, 3.22 ± 2.94 in focal NPSLE. Horizontal lines indicate median. In lower panels, correlation between CSF anti-Sm and Q albumin and that between CSF anti-Sm and serum anti-Sm were evaluated by Spearman’s rank correlation test. NPSLE: neuropsychiatric systemic lupus erythematosus; SD: standard deviation.

It has been well appreciated that damages in the BBB result in the elevation of CSF immunoglobulin [16,18,19]. We next examined Q albumin, which reflects the BBB integrity. Q albumin values in 22 control patients were 2.84 ± 1.29 (mean ± SD). Notably, there were no significant differences in Q albumin in NPSLE compared with the control group. However, among subsets of NPSLE, Q albumin was significantly elevated in ACS compared with non-ACS diffuse NPSLE (*P* = 0.0017) or with focal NPSLE (*P* = 0.0089), and also with non-SLE control (*P* = 0.0024) (Figure 4, upper panels). Accordingly, CSF anti-Sm was significantly correlated with Q albumin in NPSLE (r = 0.2373, *P* = 0.0477), although the correlation was not strong. CSF anti-Sm was also correlated with serum anti-Sm in NPSLE (r = 0.7185, *P* <0.0001) (Figure 4, lower panels). These results therefore indicate that the elevation of CSF anti-Sm in ACS is mainly a result of transudation of anti-Sm from the systemic circulation due to damaged BBB.

### Relationship of CSF anti-NR2 with CSF anti-Sm in NPSLE

**Figure 5:**
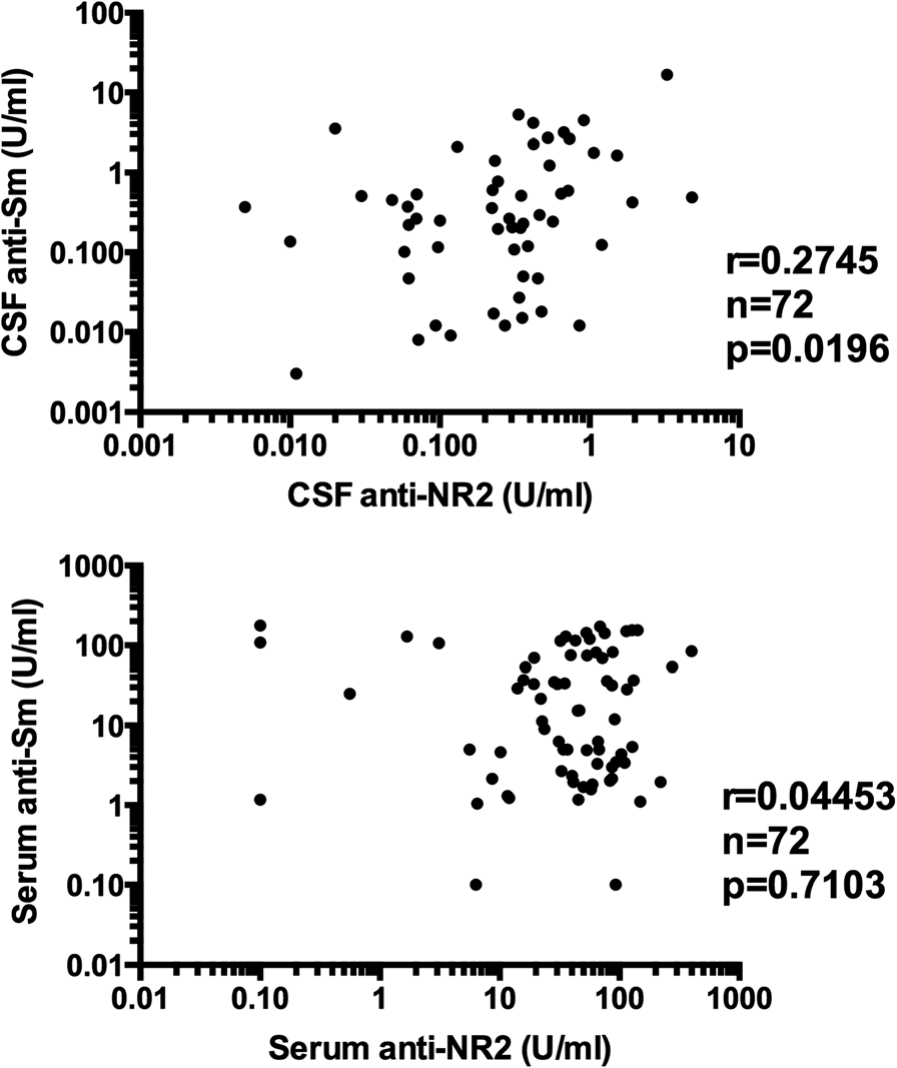
**Relationship of anti-Sm with anti-NR2 in NPSLE.** In upper panels, correlation between cerebrospinal fluid (CSF) anti-NR2 and CSF anti-Sm was evaluated. In lower panels, correlation between serum anti-NR2 and serum anti-Sm was evaluated. Statistical analysis was carried out by Spearman’s rank correlation test. NPSLE: neuropsychiatric systemic lupus erythematosus.

We have recently demonstrated that CSF anti-NR2 levels and Q albumin were significantly higher in ACS than in non-ACS diffuse NPSLE, indicating that the severity of BBB damage results in the elevation of CSF anti-NR2 [20]. We next compared CSF anti-Sm with CSF anti-NR2 in patients with NPSLE. As shown in Figure 5, CSF anti-Sm was significantly correlated with CSF anti-NR2 (r = 0.2745, *P* = 0.0196) in 72 patients with NPSLE. By contrast, there was no significant correlation between serum anti-Sm and serum anti-NR2 (r = 0.0445, *P* = 0.7103) (Figure 5 lower panel). It is thus most likely that the positive correlation between CSF anti-Sm and anti-NR2 might be due to the BBB damage. Moreover, the data suggest that the coexistence of anti-NR2 and anti-Sm in CSF might be crucial for the development of ACS.

### Binding of ant-Sm with neuronal cells

**Figure 6:**
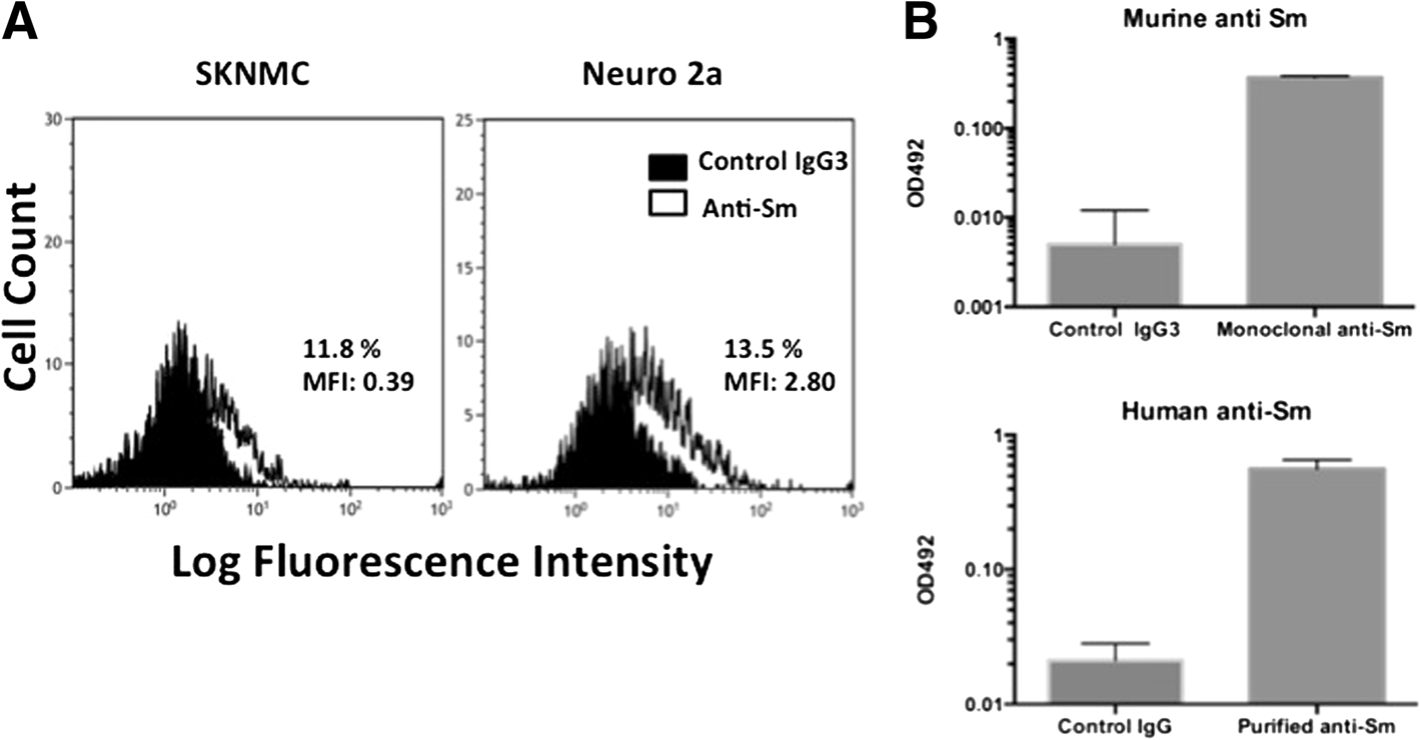
**Binding of anti-Sm to neuronal cells. (A)** After fixation with 1% paraformaldehyde, SK-M-NC cells and Neuro2a cells were reacted with murine monoclonal anti-Sm or control murine immunoglobulin G (IgG3) (5 μg/ml), followed by counterstaining with fluorescein isothiocyanate (FITC)-conjugated goat F(ab’)_2_ anti-mouse IgG and with propidium iodide (PI). The cells were then analyzed by flow cytometry. Representative FITC stainings on viable cells gated by negative staining with PI are shown. The percent positive as well as the mean fluorescence intensity (MFI) for specific anti-Sm staining are indicated. **(B)** SK-N-MC cells were seeded in wells of a flat-bottomed 96-well culture plate. After fixation with 1% paraformaldehyde, the wells were reacted with murine monoclonal anti-Sm (5 μg/ml) or human anti-Sm (50 μg/ml) purified from serum IgG fraction of an SLE patient. Murine monoclonal IgG3 (5 μg/ml) or control human IgG (50 μg/ml) of a normal healthy individual was used as controls. Bound IgG was detected with peroxidase-conjugated F(ab’)_2_ fragments of goat anti-mouse IgG or anti-human IgG. The results are expressed by the absorbance at 492 nm (OD492). Error bars indicate standard deviation (SD) of triplicate determinations.

The mechanism of anti-Sm to cause CNS damage is unknown. One possibility is that anti-Sm might directly bind neuronal cells and affect their functions. Final experiments were therefore carried out to explore whether anti-Sm might bind neuronal cells. As shown in Figure 6A, murine monoclonal anti-Sm bound to paraformaldehyde-fixed SK-N-MC cells and Neuro2a cells. Moreover, the data of cell ELISA also demonstrate that purified human anti-Sm as well as murine monoclonal anti-Sm bound to paraformaldehyde-fixed SK-N-MC cells (Figure 6B). The results therefore confirm that the epitopes recognized by anti-Sm exist on the surface of neuronal cells.

## Discussion

This study is the first to show the clinical significance of CSF anti-Sm. Thus, the results in the present study have disclosed that CSF anti-Sm is elevated in ACS, a severe form of diffuse NPSLE. By contrast, there were no significant differences in CSF anti-RNP among various subtypes of NPSLE, although both CSF anti-Sm and anti-RNP were increased in NPSLE compared with non-SLE control. It has been pointed out that anti-Sm might be involved in the pathogenesis of NPSLE [9,10]. Especially, serum anti-Sm was associated with organic brain syndrome, consisting mainly of ACS of diffuse NPSLE [11]. Consistently, serum anti-Sm appeared to be elevated in ACS in the present study as well, despite the lack of statistical significance.

Previous studies demonstrated that CSF anti-RNP was associated with NPSLE and CNS manifestations in MCTD [12]. However, the elevation of CSF anti-RNP was not specific in ACS or diffuse NPSLE, since CSF anti-RNP was also elevated in patients with neurologic syndromes, including aseptic meningitis, headache, demyelinating disorder or movement disorder [12]. Accordingly, in the present study, CSF anti-RNP was also elevated in focal NPSLE comparably to diffuse NPSLE, confirming the observation in the previous studies [12]. How anti-RNP is involved in the development of NPSLE is currently unknown. Although the possibility of induction of proinflammatory cytokines by anti-RNP was suggested [12], further studies are required to confirm this point.

Autoantibodies to NMDA receptors, a subgroup of the glutamate receptor family, have recently attracted increasing attention [5-7,21,22]. Thus, DeGiorgio *et al*. showed that injection of anti-NR2 glutamate receptor binding antibodies (purified antibodies from the sera or CSF from NPSLE patients) into mice brain resulted in apoptosis of the neuronal cells without signs of inflammation [6]. Of note, Kowal *et al*. have demonstrated that mice induced to express anti-NR2 in systemic circulation have no neuronal damage unless breakdown of the BBB takes place [7]. Importantly, we have demonstrated that monoclonal anti-Sm binds to the surface of SK-N-MC cells and Neuro2a cells, indicating that anti-Sm reacts with neurons. Moreover, the presence of greater amounts of anti-Sm in CSF was associated with ACS in the present study. Notably, the effect of anti-NR2 antibodies on neurons has been shown to be dose dependent [22]. Thus, at low concentrations they alter synaptic function, whereas at higher concentrations they can cause neuronal cell death by apoptosis [22]. It is therefore suggested that the presence of higher concentrations of anti-Sm within the CNS might cause more extensive neuronal damage, resulting in the development of ACS. The influences of anti-Sm on the function and survival of neurons are currently undetermined and need to be explored in further studies.

It has been well recognized that intrathecal production of immunoglobulin is increased in NPSLE irrespective of focal NPSLE or diffuse NPSLE [18,19]. Notably, previous studies revealed that CSF anti-RNP index was elevated in NPSLE [12]. In the present study, CSF anti-RNP index as well as CSF anti-Sm index was elevated in NPSLE compared with non-SLE control. However, there were no significant differences in CSF anti-Sm or anti-RNP index among various subsets of NPSLE. Therefore, the elevations of CSF anti-Sm levels in ACS compared with non-ACS diffuse NPSLE or with focal NPSLE cannot be accounted for by the increased intrathecal synthesis of anti-Sm.

BBB dysfunction results in the elevation of CSF immunoglobulin through the increased transudation from the systemic circulation into the CNS. In the present study, Q albumin values were not significantly elevated in NPSLE compared with non-SLE control, consistently with the previous studies [18,19]. However, among various types of NPSLE, Q albumin was significantly elevated in ACS compared with non-ACS diffuse NPSLE or with focal NPSLE in the present study, as is consistent with our recent studies [20]. On the other hand, CSF anti-Sm was significantly correlated with Q albumin, and more closely with serum anti-Sm. However, there were no significant differences in serum anti-Sm among various subsets of NPSLE and non-NPSLE, although it appeared to be higher in ACS. Taken together, these data indicate that the damage in BBB rather than the elevation of serum anti-Sm is more crucial for the elevation of CSF anti-Sm in ACS.

We have recently demonstrated that CSF anti-NR2 levels and Q albumin were significantly higher in ACS than in non-ACS diffuse NPSLE, indicating that the severity of BBB damage plays a crucial role in the development of ACS through the accelerated entry of larger amounts of anti-NR2 into the CNS [20]. The data in the present study have further disclosed that the elevation of CSF anti-Sm due to the damaged BBB is also involved in the development of ACS. In addition, CSF anti-Sm was significantly correlated with CSF anti-NR2 in NPSLE in the present study. Since there was no significant correlation between serum anti-Sm and anti-NR2, the positive correlation between CSF anti-Sm and anti-NR2 might be due to the BBB damage. More importantly, the data in the present study indicate that the elevation of both anti-Sm and anti-NR2 in CSF plays a crucial role in the development of ACS. Further studies to explore the effects of coexistence of anti-Sm and anti-NR2 on neuronal cells would be important to delineate the precise mechanism for the development of ACS.

The mechanism of damage in BBB in ACS has not been determined at present. In this regard, it is likely that several autoantibodies, such as anti-ribosomal P protein antibodies and anti-NR2 antibodies, might result in BBB damage, since they react with endothelial cells [23-25]. Notably, recent studies have disclosed that an anti-Sm autoantibody synergized with hemoglobin to enhance the secretion of proinflammatory cytokines while eliciting the increased production of monocyte migratory signals from endothelial cells [26]. It is therefore also possible that anti-Sm also might result in BBB damage. Further studies are required to explore the roles of a variety of autoantibodies in BBB damage.

In the present study, there were no differences in CSF anti-Sm or CSF anti-RNP between non-ACS diffuse NPSLE and focal NPSLE. Since a number of autoantibodies have been reported to be reactive to neurons, including anti-ribosomal P protein antibodies [27], anti-Ro antibodies [28], some anti-cardiolipin antibodies [29] and anti-NR2 antibodies [6-8], it is possible that the patterns of expression of several antibodies or their combination in CSF might be different between non-ACS diffuse NPSLE and focal NPSLE. Moreover, it is also possible that anti-ribosomal P protein antibodies, anti-Ro antibodies and some anti-cardiolipin antibodies might be also involved in the development of ACS in combination with anti-Sm and anti-NR2. Further studies are required to delineate the whole spectrum of neuron-reactive autoantibodies in CSF in order to understand the variability of manifestations of NPSLE.

A limitation of our study is that it is cross-sectional and the observations are associations and not fully causal, though the binding of anti-Sm to neuroblastoma cell lines suggests plausibility. Another limitation is the possibility that anti-Sm might have preferential properties to penetrate the CNS, leading to its higher CSF levels just as a reflection of the peripheral milieu. Therefore, it would be ideal to have an additional control of SLE patients with anti-Sm and anti-RNP in sera without CNS symptoms and study CSF anti-Sm and anti-RNP in such patients. In this regard, 5 of the 23 patients with focal NPSLE showed elevation of serum anti-Sm over 50.0 U/ml without psychiatric manifestations. When compared with 5 patients with ACS diffuse NPSLE who showed almost the same values for serum anti-Sm, the 5 patients with focal NPSLE showed significantly lower CSF anti-Sm values (data not shown). Therefore, it is strongly suggested that elevation of CSF anti-Sm might be causal for ACS diffuse NPSLE.

## Conclusions

The current studies disclosed that CSF anti-Sm levels and Q albumin were significantly higher in ACS than in non-ACS diffuse NPSLE (anxiety disorder, cognitive dysfunction, mood disorder and psychosis) or in focal NPSLE, whereas there was no significant difference in CSF anti-Sm index among the 3 groups. CSF anti-Sm was significantly correlated with Q albumin and with CSF anti-NR2 in NPSLE. Finally, anti-Sm has been demonstrated to react with neuroblastoma cell lines. These results indicate that the elevation of CSF anti-Sm and anti-NR2 due to BBB damage plays a critical role in the pathogenesis of ACS, a severe form of diffuse NPSLE. More importantly, the data indicate that anti-Sm is yet another autoantibody with presumed neural toxicity, but might not be the last.

## Abbreviations

ACR: American College of Rheumatology
ACS: acute confusional state
anti-NR2: antibodies reactive with NMDA receptor NR2 subunit on neuronal cells
BBB: blood-brain barrier
CNS: central nervous system
CSF: cerebrospinal fluid
ELISA: enzyme-linked immunosorbent assay
HSA: human serum albumin
IgG: immunoglobulin G
MCTD: mixed connective-tissue disease
MFI: mean fluorescence intensity
NMDA: N-methyl-D-aspartate
NPSLE: neuropsychiatric systemic lupus erythematosus
PBS: phosphate-buffered saline
PI: propidium iodide
